# How can technology be used to support communication in palliative care beyond the covid-19 pandemic: a mixed-methods national survey of palliative care healthcare professionals

**DOI:** 10.1186/s12904-024-01372-z

**Published:** 2024-02-14

**Authors:** Sarah Stanley, Anne Finucane, Anthony Thompson, Amara Callistus Nwosu

**Affiliations:** 1 Marie Curie Hospice Liverpool, Liverpool, L25 8QA United Kingdom; 2 Clinical Psychology, University of Edinburgh, Edinburgh, United Kingdom; 3 St Helens and Knowsley NHS Foundation Trust, Prescot, United Kingdom; 4 Lancaster Medical School, Lancaster University, Lancaster, United Kingdom; 5 Liverpool University Hospitals NHS Foundation Trust, Liverpool, United Kingdom; 6 Liverpool John Moores University, Liverpool, United Kingdom; 7 Marie Curie Hospice Edinburgh, Edinburgh, United Kingdom

**Keywords:** Technology, Communication, Palliative care, Healthcare, COVID-19, Pandemic

## Abstract

**Background:**

Developments in digital health have the potential to create new opportunities for healthcare professionals support delivery of palliative care. Globally, many palliative care professionals used digital health innovations to support communication with staff, patients and caregivers, during COVID-19 pandemic. However, there is limited data about the views of palliative care professionals of using digital health to support communication during the pandemic. We aimed to describe how palliative care professionals used technology to support communication (multidisciplinary team working, education and with patients and family caregivers) during the COVID-19 pandemic.

**Method(s):**

UK based palliative care healthcare professionals completed an electronic questionnaire to describe their use of digital health, during the COVID-19 pandemic, to support (1) communication within the multidisciplinary team (MDT), (2) education and (3) to support communication with patients and carers.

**Results:**

Two hundred and thirty-four palliative care professionals participated. Most (*n* = 227, 97%) described an increase in their use of digital health, to support communication, since the start of the COVID-19 pandemic. We identified benefits and challenges for digital health communication, which we summarised into themes, including ‘a new way of working’, ‘developing a new approach to learning’ and ‘impacting care’.

**Conclusion(s):**

Since the pandemic, palliative care professionals have increased their use of digital health to support communication in clinical practice. We have identified facilitators and barriers for future practice. Further work should identify the levels of support needed for organisations to ensure that digital health interventions are meaningfully used to help palliative care professionals effectively communicate with patients, caregivers and staff.

**Supplementary Information:**

The online version contains supplementary material available at 10.1186/s12904-024-01372-z.

## Key Statements

What is already known about the topic?

- During the COVID-19 pandemic, palliative care teams worldwide have used digital health to communicate within multi-disciplinary teams, to deliver education and to communicate with patients and family caregivers.
- There is limited evidence of how digital health has been used within the palliative care speciality.

What this study adds

- Identifies learning points to support the use of technology beyond the pandemic.
- Describes recommendations to support adoption of technology in clinical practice.

How this study might affect research, practice or policy

- Organisations should develop and adopt models of care which use technology to support communication in palliative care.
- Organisations should review practical, governance and resource issues which are necessary to ensure that technology is used safely and effectively.
- Further research is required, across different international and palliative care settings, to explore different cultural attitudes to technology, and the experiences of healthcare professionals who are unfamiliar with using technology in practice.
- Researchers should also explore patient and caregivers’ views about the use of technology to support communication.

## Introduction

Many healthcare professionals and organisations have used digital health; describing technologies which use computing platforms, connectivity, software, and sensors for health care and related purposes, such as communication[1, 2]. These technologies have been used to facilitate virtual communication, and reduce the risk of viral transmission, due to the global COVID-19 pandemic [3–5]. During the pandemic, palliative care teams worldwide have used digital health (for example, video calls, email and text messaging) to communicate within MDT’s, [6, 7] to deliver education [8, 9] and to communicate with patients and family caregivers [10, 11].

There is a lack of data about the use of digital health to support people with palliative care needs [12]. This is significant, as people with palliative care needs have more specialist care needs compared to other medical and surgical patients [13, 14] so data describing the practicalities of digital health use may not translate to palliative care populations [2, 15]. Currently, there is limited data about the experiences of palliative care professionals and how they have used technology to support communication during the COVID-19 pandemic [2]. Understanding the benefits, challenges and opportunities of digital health, as identified by palliative care professionals, will help organisations to develop the models of care needed to help palliative care professionals use technology meaningfully to support communication.

### Aims

To describe the experiences of UK based palliative care professionals who used technology to support communication for multidisciplinary team working, education and with patients and family caregivers, during the COVID-19 pandemic.

The objectives were:

- To identify factors where the use of technology can support palliative care communication beyond the COVID-19 pandemic.
- To describe factors that support adoption of technology to enhance communication in palliative care.

## Methods

### Study design and aim

This was an electronic questionnaire of UK based palliative care healthcare professionals which looked to explore the use of digital health during the COVID-19 pandemic. The study is reported adhering to the Checklist for Reporting Results of Internet E-surveys [16].

### Participants

UK based healthcare professionals who were working in a palliative care team during the COVID-19 pandemic from March 2020 onwards. We looked to include healthcare professionals of all disciplines (e.g., medical, nursing, social work, therapy, pastoral) caring for palliative patients.

Inclusion criteria were as follows: Palliative care healthcare professionals, working in a palliative care setting, working during the COVID-19 pandemic beginning in March 2020.

### Questionnaire development

We used data from a published Delphi study which described research priorities of technology in palliative care [2] to inform the questionnaire development. We conducted workshops in the Marie Curie Hospice Liverpool, to identify the views of multidisciplinary staff, to gain perspectives of the broad areas of how technology has been used during the pandemic (Appendix 1). We used this information to further develop the non-randomised open and closed questionnaire items.

We asked participants to answer questions about their experiences of using technology in the context of:

1. communication within the MDT, including the reasons they used technology and their experience of remote sessions.
2. for education, including the type of sessions they were involved with and their preferred methods of learning.
3. to support communication with patients and caregivers, to determine examples of how technology was used (e.g., outpatient appointments, calls between patients and relatives), and determine facilitators and barriers to using technology in practice.

We used Microsoft Forms (https://forms.microsoft.com) to develop the open web questionnaire. The questionnaire mostly consisted of multiple-choice questions; participants were able to provide further free text responses for some questions (Appendix 2). The questionnaire had a maximum of 36 questions across 7 pages (depending on responses), and we anticipated that it would take 15 min to complete. All questions were made mandatory, with the exception of free text responses, and where appropriate respondents were given the opportunity to provide a non-response such as ‘not applicable’. We asked participants to provide information about demographics, device type and software platforms used. Participants were able to review and change their answers using the back button. We defined communication ‘technology’ as any product that stored, retrieved, manipulated, transmitted, or received information electronically in a digital form (e.g., personal computers, tablets, email, smart assistants).

### Piloting

Prior to use, we piloted the questionnaire (two clinicians and two academics) to ensure that the questions were clear, and the instructions were simple to understand.

#### Recruitment

The voluntary (non-incentivised) questionnaire was disseminated through professional networks, social media, and email (Appendix 3). Data were collected between May and June 2021. We sent an email to invite potential participants to participate in a short questionnaire (Appendix 3), which included a participant information sheet and the consent form (Appendix 2). Participants were asked to read the participant information sheet (Appendix 4) which provided details on data protection and contact details for investigators. We promoted the questionnaire on Twitter (and other social media) using posts which included palliative care related hashtags (e.g., #hapc, #hpm, #palliativecare), and a weblink to access the relevant documents for those wishing to participate.

We received 241 survey responses. Via contact and demographic information, we identified one duplicate which was removed, and the first completed survey was retained. Six (*n* = 6) participants reported that they had not used technology during the COVID-19 pandemic and therefore did not complete the survey. These were not included in data analysis.

### Data analysis

We used Microsoft Forms to analyse data and report frequencies. We used the Braun and Clarke six-phase process [17] to analyse free-text data using inductive thematic analysis. We coded responses line by line and identified descriptive themes. We analysed 450 free text responses, and we identified emerging themes. Participants commented on the use of technology to support communication across the three different areas (1) Use of technology to support communication within the MDT, (2) Use of technology to support education and (3) Use of technology to support communication with patients and carers as defined in our questionnaire.

## Results

### Sample characteristics

234 palliative care healthcare professionals completed the survey. Most respondents were female, based in England and working as a doctor or nurse. Most participants were working in community or a hospice inpatient unit (Table 1).

**Table 1:** Demographic data *n* = 234 (%)

Gender	
Female	207 (88.4%)
Male	27 (11.5%)
Area of the UK	
England	203 (86.8%)
Scotland	14 (5.9%)
Wales	11 (4.7%)
Northern Ireland	6 (2.6%)
Place of work	
Community service	75 (32.0%)
Hospice inpatient unit	73 (31.2%)
Hospital advisory team	39 (16.7%)
Hospital inpatient ward	13 (5.6%)
Other	34 (14.5%)
Profession	
Doctor	106 (45.3%)
Nurse	60 (25.6%)
Healthcare Assistant	27 (11.5%)
Social worker	8 (3.4%)
Occupational therapist	6 (2.6%)
Physiotherapist	6 (2.6%)
Pharmacist	2 (0.9%)
Other	19 (8.1%)

**Fig. 1:**
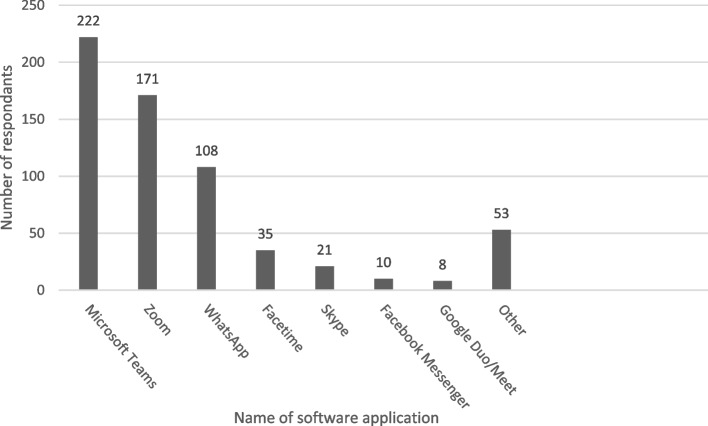
Software applications used

Most respondents (227/234, 97.0%) said they had used technology more, to communicate, since start of the COVID-19 pandemic. Microsoft Teams and Zoom were the most frequently used software applications (Fig. 1).

### Emerging Themes

From the qualitative data we identified three major themes, with subthemes, which are presented in Table 2.

**Table 2:** Overview of qualitative theme

Key areas of interest	Emerging main theme	Sub-themes	Quotes from data
Using technology to support communication in the Multidisciplinary Team	A new way of working	• Collaboration • Personal skill development	*‘We have organised MDT meetings *via* teams with professionals from several organisations – this would have been impossible to set up in person so probably wouldn’t have happened pre-pandemic’. ****Respondent 11, Doctor*** *‘Using video conferencing for joint hospice/community/hospital MDT has facilitated increased participation and reduced the time commitment from travelling’. ****Respondent 21, Nurse***
Using technology to support Education	Developing a new approach to learning	• Accessibility • Convenience • Economic benefits	*‘Being able to attend remotely has allowed for more learning to be made use of due to flexibility and being opportunistic when 'quiet'.’ ****Respondent 34, Doctor*** *‘I used to think that face to face was always best, but in this last year I've come to realise that joining conferences, *etc. *remotely opens up access to experts in a whole new way and is so much more time efficient, not having to take whole days out and do loads of time-consuming travel—love it (and NEVER thought I'd say that!!!)’. ****Respondent 121, Nurse*** *‘With virtual training I've been able to attend training that I otherwise wouldn't have been able to go to, because they'd be too far away, too expensive, or take up too much time when travelling's included’. ****Respondent 2, Doctor***
		• Social isolation • Fatigue • Technical issues	*‘Virtual (sessions) alone can lead to a feeling of isolation when learning and doesn't always facilitate questions and answers’ [sic]. ****Respondent 163, Nurse*** *‘I like to engage with the teaching and although it is possible to ask questions and become more involved *via* Zoom, for example, it is also possible to switch off and be less committed to the learning event due to being on ones own, being distractable, and not being observed*’. ***Respondent 143, Clinical Psychologist*** *‘Technology tends to have “computer said no” errors and mishaps, along with questionable hospital WiFi. Often too many people in the office, even with masks on, causes plenty of feedback. Still difficult for people to get their heads around, in terms of how to operate online virtual sessions’. ****Respondent 100, Pharmacist***
Using technology to support communication in with patients and carers	Impacting care	• Psychological wellbeing • Physical wellbeing • Enhanced care	‘*At home quiz—the element of the socialising of this group has been helpful for our patients who perhaps feel quite isolated. we get on average 17 patients a week over 5 sessions (1 h long)’. ****Respondent 20, Doctor*** *‘Having technology available to facilitate communication with patients and their loved ones when visiting wasn’t possible made some extremely difficult situations more bearable for many dying patients and their families’. ****Respondent 148, Counsellor***
		• Technological barriers • Privacy • Concern of less effective care	*‘IT can only work with those patients who have the technology available and can use it. On some occasions technology failed and the session had to be aborted’ ****Respondent 14, Nurse*** *‘How he presented over video was so much different to when he needed to come and see me face to face. He appeared frail and watching him walk from the waiting room to my room gave me a lot of information I was not privy to before. I was amazed at how much I had missed by videoing him!’ ****Respondent 122, Doctor***

### Use of technology to support communication in the multidisciplinary team

#### Quantitative findings

Most palliative care healthcare professionals used technology to support communication with MDT members (191/234, 81.6%). Staff said that they used technology to reduce face-to-face interaction whilst physically present at work (204/234, 87.2%), to virtually attend meetings with people external to of their primary workplace (198/234, 84.6%), to provide opportunities to work flexibly from home (151/234, 64.5%) and to enable longer term home working for those needing extended self-isolation periods (e.g. clinically vulnerable individuals and those with caring responsibilities) (65/234, 27.7%).

#### Qualitative findings

*A new way of working.* Free-text comments provided greater insight into how palliative care healthcare professionals described a ‘new way of working’ using technology to support communication within the multidisciplinary team. Participants’ free-text responses also demonstrate that technology has been used to support carers (for example when caring for young children or vulnerable relatives), to reduce home visits and car travels.

*Collaboration.* Respondents said that they had used technology to improve their ability to virtually collaborate, and attend meetings with, multidisciplinary healthcare professionals who work in different settings.

*‘The pandemic has pushed forward the use of technology for more effective communication in all aspects of the job, particularly enhancing communication with MDTs enabling us to meet with GPS, district nurses and other members of the team more regularly and reduced the travelling time.’ ****Respondent 40, Doctor***

*Personal skill development.* Respondents said that the requirement to use technology to support communication in their practice has helped them to develop new skills, which contributed to a personal sense of achievement.

*‘I have noticed that the need to use technology during the pandemic has allowed people who are not so confident to learn how to use it and feel more able to assist a patient with a video call or join in on group meetings without feeling anxious about it.’ ****Respondent 85, Nurse***

### Use of technology to support education

#### Quantitative findings

**Fig. 2:**
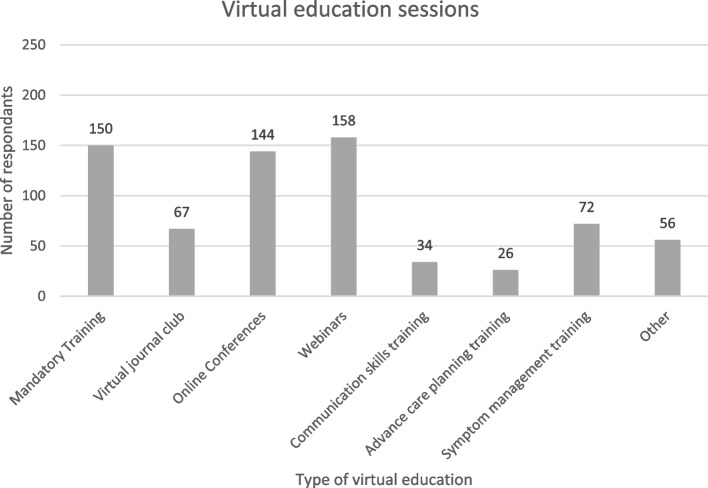
Virtual education sessions accessed

Most respondents had participated in virtual education during the pandemic (225/234, 96.2%), which included webinars, online conferences, and online mandatory training. Respondents had used technology to virtually participate in local education, such as journal club, communication skills and symptom management training (Fig. 2). Most (183/234, 78.2%) said that their learning preference was a combination of virtual and face-to-face learning.

#### Qualitative findings

*Developing a new approach to learning.* Respondents said that they enjoyed aspects of both face-to-face and virtual approaches. Several respondents said that a combination of face-to-face and virtual approaches would benefit future learning. We identified six subthemes which are described below.

*Accessibility.* Respondents said that virtual education sessions can provide more options for people to attend meetings, which can help to increase audience participation to include people who may otherwise not have been able to attend. Respondents also said that virtual sessions have improved options to support continued professional development.

*I have struggled to go to face to face education away from home so this year has been easier as the providers have flexed their provision.’ ****Respondent 41, Chaplain***

*Convenience.* Participants valued the flexibility of attending virtual sessions and the ability to catch-up, or rewatch, sessions at their convenience.

*‘Virtual education has enabled greater participation due to challenges of travelling distances to attend training but misses out on the valuable social aspects and networking gained by face to face teaching.’ ****Respondent 32, Nurse***

Many described how using virtual education has helped them to attend sessions as they manage other commitments, such as carer and childcare responsibilities.

*‘As a single mother it's a huge challenge to be out of the house even for an extended day. So, whilst I prefer F2F [sic] education, this only works for me if I can get there and back during a normal school day. For local, and short sessions it's great, but I usually have to miss out on the bigger / longer / further away events altogether. This year has been great, as the on-line / virtual opportunities have been fantastic, and I've been able to attend loads of events that I would never have been able to get to in person.’ ****Respondent 19, Doctor***

*Economic benefits.* Respondents said that virtual education was better for the environment than face to face, as this helped to reduce work-related car travel. Several participants said that virtual education can potentially reduce travel costs and contribute to a better work-life balance.

*‘I really like the accessibility of not having to travel & being able to fit more in* + *save money* + *save carbon.’ ****Respondent 138, Social Worker***

*Social Isolation.* Respondents said that feeling isolated, when participating in remote learning, was a barrier to learning. Several said that reduced social interaction, during sessions, made it difficult to engage in educational activity. Some respondents also described how social isolation was exacerbated by reduced opportunities for networking and peer support.

*‘Face to face meetings allows for more socialising and informal peer support.’ Respondent ****80, Healthcare Assistant***

*Fatigue.* Respondents said that the increased amount of video calls caused fatigue which was a barrier to learning. Some respondents said that distractions in their virtual learning space also reduced the capacity to learn.

*‘I like human interaction as an active learner. Too much screen time is exhausting and you lose a lot of non verbal communication and intuitive sensing that comes with being in the same space as other people.’ Respondent ****81, Doctor***

*Technical issues.* Respondents said that technical challenges with technology were a common barrier to learning.

*‘I've missed doing things in person and the technology doesn't always work as desired!’ ****Respondent 2, Doctor***

Participants said that a face-to-face teaching component was essential for the following eight educational activities: Cardiopulmonary resuscitation, ethics training, leadership training, communication skills training, conferences, first aid training, manual handling training and clinical mentorship/supervision – in which healthcare professionals are given an opportunity to reflect in a supportive environment.

*‘I feel that for some education it is sensible for virtual training but for others e.g. CPR then you need the face to face*.’ ***Respondent 12, Doctor***

*‘Some mandatory training necessitates face to face sessions such as Personal Handling to assess correct technique.’ ****Respondent 60, Doctor***

Most respondents described the need for palliative care to adopt *‘a blended approach to learning’*, where technology is used alongside face-to-face teaching. Respondents described this positively, as a way to reduce non-essential travel, whilst maintaining face-to-face contact as desired.

*‘A blended approach with technology supporting communication has been helpful but there remains a clear role for face-to-face communication - whether that is MDT, education or patient and carer support.*’ ***Respondent 37, Doctor***

### Use of technology to support communication with patients and caregivers

#### Quantitative findings

Most respondents said that they worked with patients and caregivers as part of their job (218/234, 93.2%), and the majority (174/234, 74.4%) had used technology to support communication. Of these responses, the most common reason for using technology was to provide information to family caregivers (123/174, 70.7%). Several respondents said they used technology to arrange personal calls between patients and relatives (108/174, 62.1%) at specific circumstances, such as when the patient was entering the dying phase, when intensive therapies were withdrawn, when visiting restrictions were implemented, and to support spiritual care. Over half of respondents (98/174, 56.3%) had used technology to support outpatient appointments for several reasons. For example, to deliver psychological therapy, initial assessments, virtual home visits, emotional and practical support, and advance care planning discussions. Several respondents (45/174, 25.9%) reported that they had used technology to facilitate group activities for patients and caregivers (Table 3).

**Table 3:** Examples of group activities facilitated using technology

Care planning meeting for inpatient	Music therapy
Exercise group	Tai Chi
Social group	Mindfulness
Bereavement support	Singing group
Craft group	Bingo
Relaxation group	1–2-1 complementary therapy
Yoga	Sibling support group
Coffee morning	Family fun activities
Symptom control and peer support	Young adult support group
Chair based exercises	Parents group aimed at parents from minority ethnic backgrounds
Reminiscence rooms: sharing memories	Virtual cooking group

#### Qualitative findings

*Impacting care.* In our free-text data analysis of the ‘technology to support patients and caregivers’ category, we identified one major theme, *‘impacting care’*. Six subthemes were identified in this category and are described below.

*Psychological wellbeing.* Participants described examples of virtual group activities and how these sessions had helped to support patient and carer wellbeing, as they provided patients with structure and social interaction during covid-related restrictions. Respondents commented on the perceived success of these practices, for example virtual quiz groups were deemed to work better than patient-led peer support group sessions.

*‘Seated tai chi courses via Zoom. Feedback has been largely positive about being able to engage in an activity and be part of a group during this time.’ ****Respondent 32, Nurse***

*Physical wellbeing.* Non-medical staff (e.g., physiotherapists and occupational therapists) identified how they were able to use the group activity to identify physical changes in a patient’s condition to identify whether medical review where needed.

*‘Exercise group keeps patients motivated. Has also allowed staff to monitor patients and identify health issues which we have managed appropriately and timely.’ ****Respondent 16, Senior Care Coordinator***

*Enhanced care.* Many respondents said that technology had a positive impact on patient care, as clinicians used the technology to support their patients’ care preferences (for example, providing the option of a telephone consultation). Several respondents highlighted how technology had helped to improve options for patients, in particular video calls, which helped to facilitate connection when face-to-face visits were not possible.

*‘A patient wanted to share Holy Communion with his wife. At that time visiting was restricted so we did communion over the phone with her. I have also prayed with a family and patient over the phone.’ ****Respondent 41, Chaplain***

*Technical barriers.* Participants reported how technical issues with the technology (e.g., WiFi connection, poor video and audio quality) contributed to challenges to delivering and facilitating group sessions.

*‘Technological issues can be a barrier, for instance microphone not picking up everyone in a room.’ ****Respondent 59, Doctor***

*Privacy concerns.* Some respondents highlighted issues about the lack of privacy within households and how some patients, and caregivers, may not participate in virtual group activity compared to face-to-face sessions.

*‘The carers group was paused as carers did not want to talk about their situation whilst their loved ones were in the same house and could potentially hear.’ ****Respondent 32, Nurse***

*Concern of less effective care.* Several respondents were concerned that virtual care provided ‘less effective’ care than traditional face-to-face models of communication. Respondents said that video calls (compared to face-to-face contact) may be distracting for patients, did not offer the same level of support and reduced openness between patients and clinicians. Two participants described face-to-face consultations as ‘the gold standard of care’. Many respondents said they believed that the use of technology increased isolation for some patients and caregivers and that physical symptoms were missed on video consultations.

*‘Whilst technology has had its place during the pandemic I would not want to see a drive for it to replace face to face consultations with patients and families as I think I this remains the gold standard.’ ****Respondent 28, Doctor***

## Discussion

### Summary

This study describes positive and negative factors that are associated with using technology to support palliative care communication during the COVID-19 pandemic in the UK. These factors include:

1. For communication within the multidisciplinary team (MDT): Collaboration and personal skill development.
2. For education: Accessibility, convenience, economic benefits, social isolation, fatigue and technical issues.
3. To support communication with patients and caregivers: Psychological wellbeing, physical wellbeing, enhanced care, technological barriers, privacy and concern of less effective care.

By addressing the use of technologies in these three areas we have been able to identify how digital technologies might continue to support communication beyond the COVID 19 pandemic, describing factors which support its use for the future. This is one of a few studies which describes the experiences of palliative care professionals of using technology for communication in clinical practice during the COVID-19 pandemic.

### What this paper adds in relation to previous work

We observed that a high proportion of palliative care healthcare professionals (174/234, 74%) had increased their use of technology to support communication in clinical care, showing the willingness of palliative care healthcare professionals to adapt their way of working in order to provide high quality care [12, 18, 19]. Most respondents used a desktop or laptop to communicate during the pandemic, which is a change from pre-pandemic where mobile devices were most commonly used [20, 21]. This change is possibly due to the staff needing to use desk-based video conferencing technology for work, in addition to the need for more intensive computer-based tasks, which were easier to conduct with a desk-based computer.

Our participants believed that the incorporation of digital health in palliative care practice will increase in the future, with priorities for digital health technologies in palliative care becoming increasingly important [2]. The pandemic has rapidly accelerated adoption of these technologies and healthcare professionals have adapted to incorporating them into their practice [2, 14, 22]. We identified several facilitators (e.g., accessibility, convenience and psychological wellbeing) as well as barriers (e.g., technological barriers, social isolation and a lack of confidence) to using technology to support communication. Our findings are consistent with previous work which highlights the technical, practical and ethical challenges which are associated with the adoption of digital health in palliative care [2, 18]. Findings from this study emphasise the potential to take a hybrid approach to future working in which digital technologies could be used alongside usual practice in order to enhance communication in palliative care.

Our work also highlights that a large proportion of palliative care healthcare professionals have participated in virtual learning since the start of the pandemic, which is consistent with research in other healthcare disciplines [23, 24]. Further, research has demonstrated the value of virtual learning in palliative care healthcare professionals in community settings [25]. We identified benefits of virtual education, such as convenience and accessibility; however, we identified that it is important to consider the most appropriate method to deliver sessions, as some will be better suited to virtual, face-to-face or hybrid approaches. One solution to this could be a blended learning approach (i.e., a mix of virtual and face to face education), which has shown to be effective and create a positive learning experience amongst medical and nursing students [26, 27].

Globally, COVID-19 health restrictions meant that palliative care services were required to quickly implement technological solutions to provide care, despite pre-pandemic work which highlighted potential challenges of its use in practice [28–30]. Although many areas have resumed face-to-face services as the pandemic progressed, it is possible that technological innovations, implemented by palliative care services during the pandemic, will persist to provide care options for patients and caregivers [5, 31]. Therefore, it is important to consider staff, patients and caregivers’ preferences for virtual or face-to- face interaction across settings.

### Limitations

The demographics of participants may reduce the generalisability of our data. For example, as we did not collect data on participant’s age, culture or ethnicity, we do not know about how these factors may have shaped the views, cultural norms and preferences of respondents. The survey was distributed across the four nations of the United Kingdom, but most responses were from England, which may mean that our results are not generalisable to the entire UK or other international healthcare settings.

It is possible that people who were less familiar with using technology did not participate in the study due to the requirement to complete an electronic questionnaire, which means that the study findings may not represent the views of the wider palliative care workforce. Consequently, it may be necessary to explore the views of palliative care professionals who are not be comfortable with using technology in practice (potentially using non-technological forms of data capture) to capture their views and experiences. It is possible that respondents may have had different interpretations of the terminology describing technology, which could have caused variation in respondents’ answers to survey questions.

### Implications for policy and practice and research

It is important for organisations to develop and adopt models of care which use technology to support communication in palliative care. Organisations should review practical, governance and resource issues which are necessary to ensure that technology is used safely and effectively. Research is required, across different international and palliative care settings, to explore different cultural attitudes to technology and the experiences of healthcare professionals who are unfamiliar with using technology in practice. Researchers should also explore patient and caregivers’ views about the use of technology to support communication.

We have highlighted approaches to help practitioners use this technology meaningfully in the future and adopt the technology in practice. We have identified learning points and factors which can support adoption of technology in practice (Table 4).

**Table 4:** Recommendations for palliative care organisations to support the use of technology, for communication, in practice

Learning points to support the use of technology beyond the pandemic	Recommendations to support adoption of technology in practice
- **Developing a matrix of activities:** which are most suited to online, face to face and which should take a hybrid approach	- **Environment:** ensuring technology and room space is appropriate
	- **Training:** help and support for patients and family caregivers to use equipment
- **Improving access:** Considering how technology fits in with local services	- **Governance:** ensuring appropriate policies are in place
	- **Patient liaison teams** For example, in hospitals to set up video calls with patients and families
- **Staff wellbeing:** Using technology to improve work/life balance for staff	
- **Enhancing models of care:** Using technology for future engagement and accessibility	- **Concentrate on the person and not the technology** Ensure individual preferences are considered
	- **Partnership with other organisations to share resources** This should encourage individuals to adopt technological interventions (for example through joined up journal club or online conferences)

## Conclusions

The use of digital technologies to support communication in palliative care is increasing. It is important that we consider how technology can be used effectively in the future by addressing the facilitators and barriers highlighted in this study. Further work should identify the levels of support needed for organisations to ensure that digital health interventions are meaningfully used to help palliative care professionals effectively communicate with patients, caregivers and staff.

### Supplementary Information

**Additional file 1. **

## Data Availability

All electronic data was stored on a secure password-controlled drive, and for 10 years after publication of the results before being confidentially deleted.

## Abbreviations

MDT: Multidisciplinary team
