# Supplementary material for: How can technology be used to support communication in palliative care beyond the covid-19 pandemic: a mixed-methods national survey of palliative care healthcare professionals
Source: BMC Palliat Care. 2024 Feb 14;23:40. doi: 10.1186/s12904-024-01372-z (PMC10865684; doi:10.1186/s12904-024-01372-z)
Supplement: Supplementary file 1 — Additional file 1. [file 12904_2024_1372_MOESM1_ESM.docx]

**SUPPLEMENTARY MATERIAL**

**Appendix 1. Use of technology in a hospice multidisciplinary team**

Multidisciplinary Team

Handovers – Use of Microsoft Teams due to reduced capacity in rooms (social distancing)

Use of Microsoft Teams for interface (meeting between different organisations)

Both have improved over time – becoming familiar with set up of virtual meetings

Confidence in speaking in meetings

Speaker quality poor on laptops

No camera on desktop computer

Education

Able to attend local education more often (e.g. journal club)

Able to attend more external education

Difficult to hear at times if not in the room (especially in bigger rooms)

Sometimes people are ‘put off’ participating in discussion virtually

All online during pandemic

Lacking opportunities for reflection

Patients and Carers

Facetime and What’s app calls from own devices

I pads provided by hospice: easy to use for most staff, most patients liked these and keeping connected with family.

When visiting restricted: families felt they were bothering staff by phoning

Phone calls same length, volume of calls increased

Anxieties (staff) over phone calls when patient had deteriorated

Technological barriers (e.g. patient unable to log on or have access to technology)

Sometimes generation and age of patient is a barrier

Patients thankful for social aspect of groups

Difficult to not be with patients during difficult conversations

Able to assess patient’s condition over video

**Appendix 2. Healthcare Professionals Questionnaire**


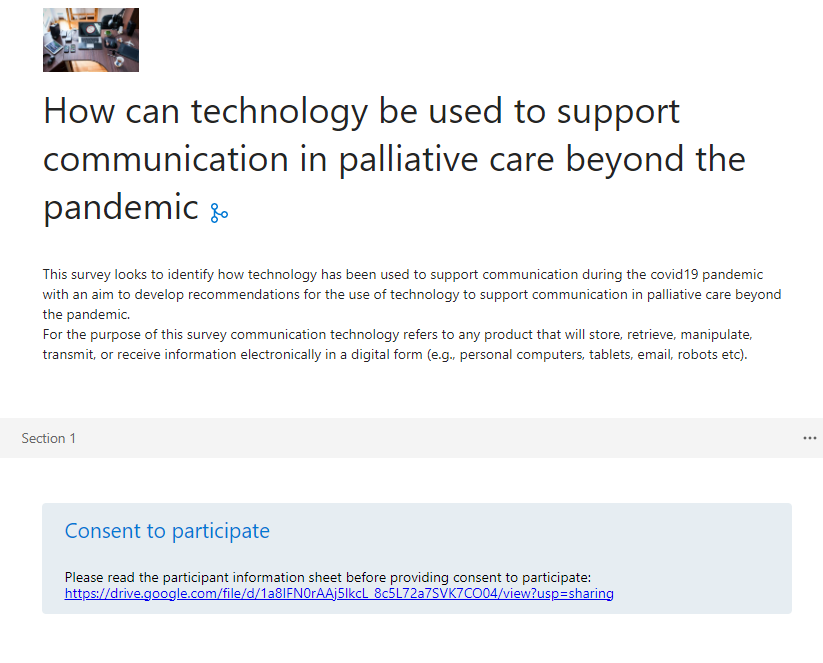


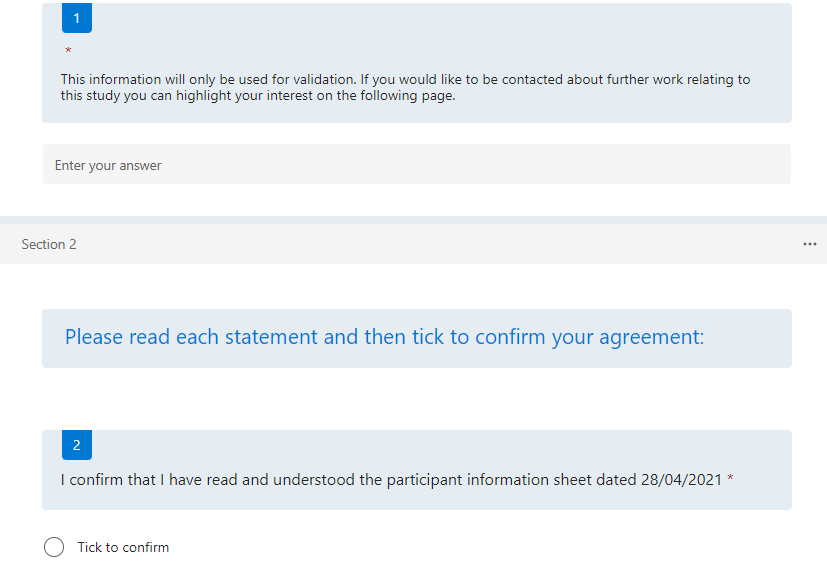


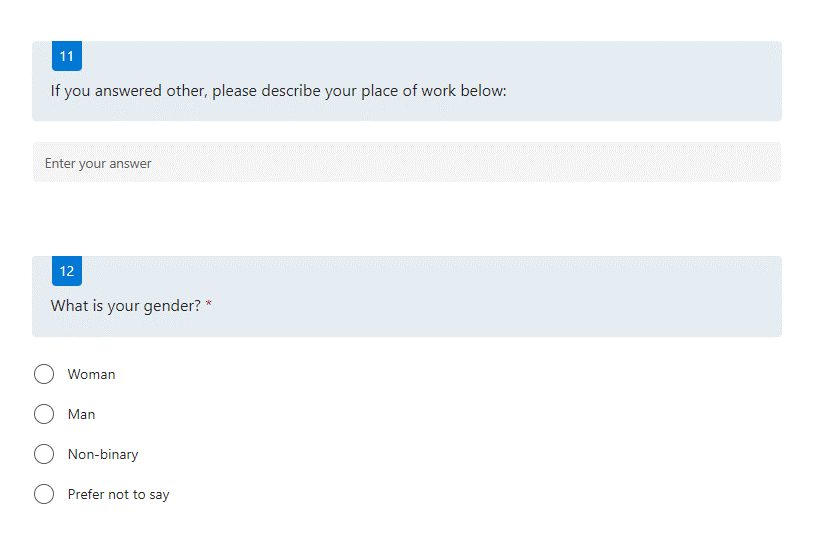

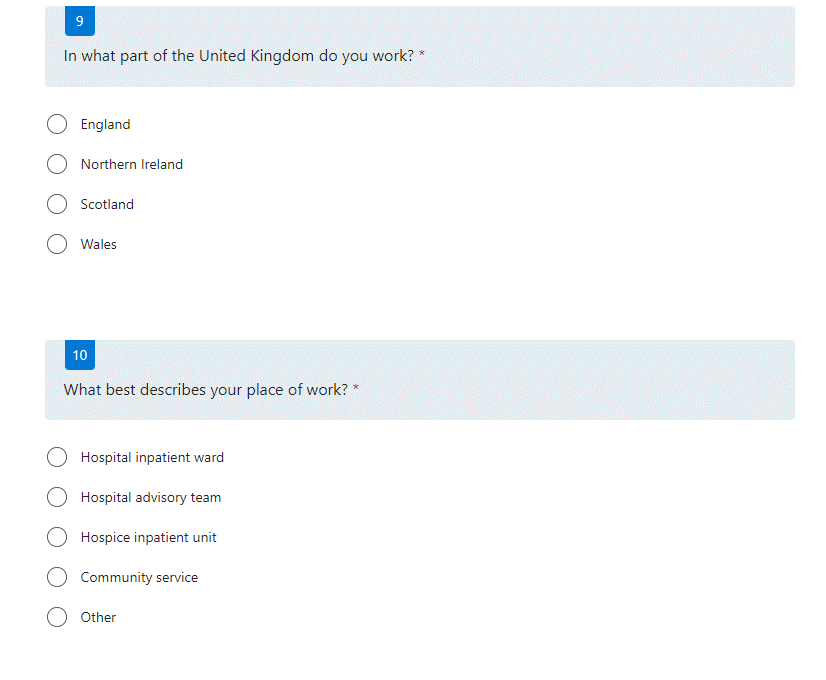

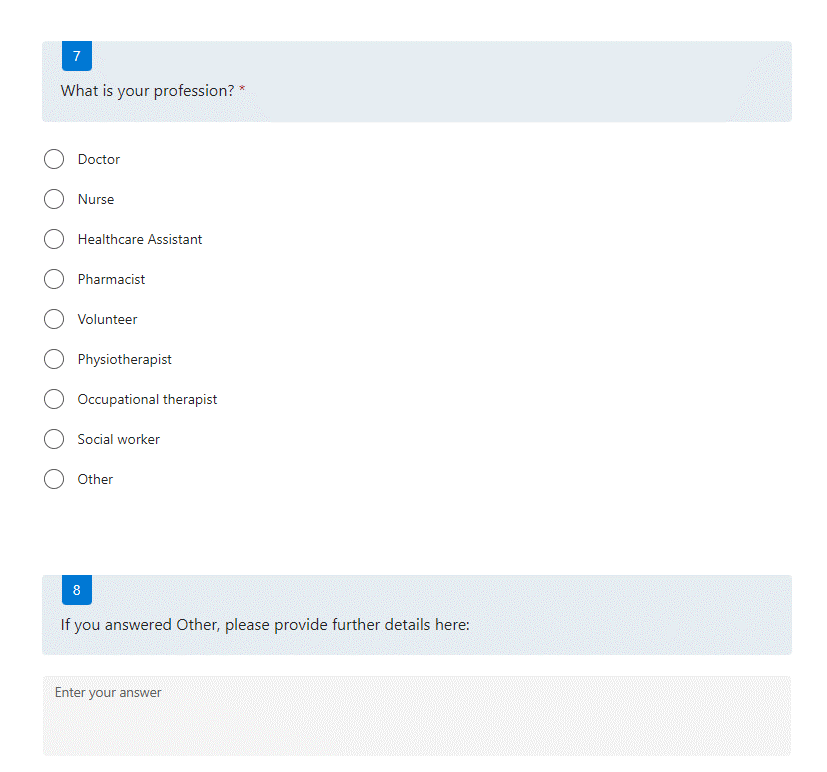


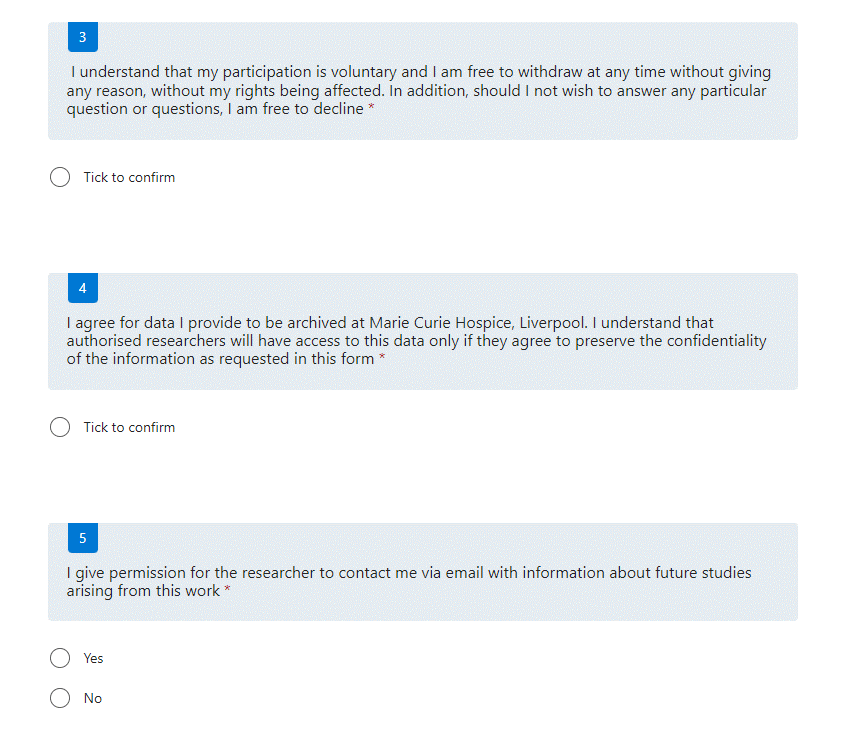


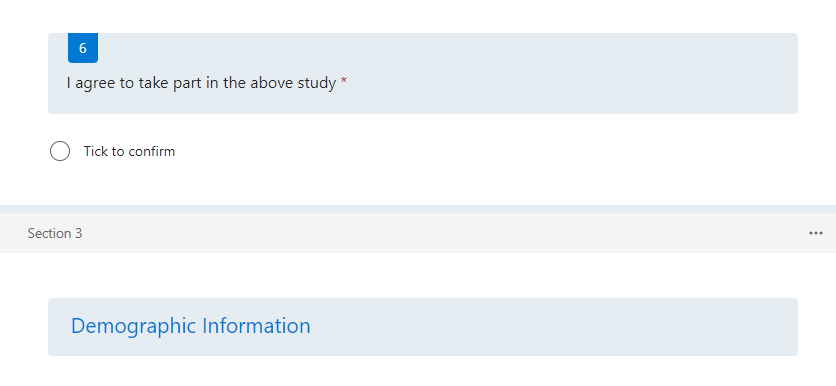


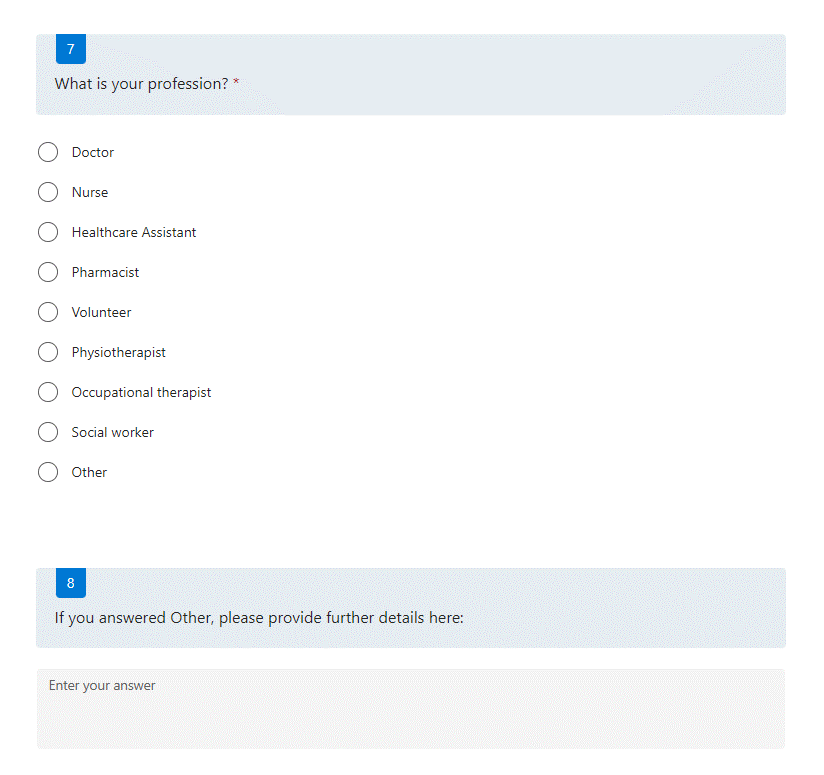


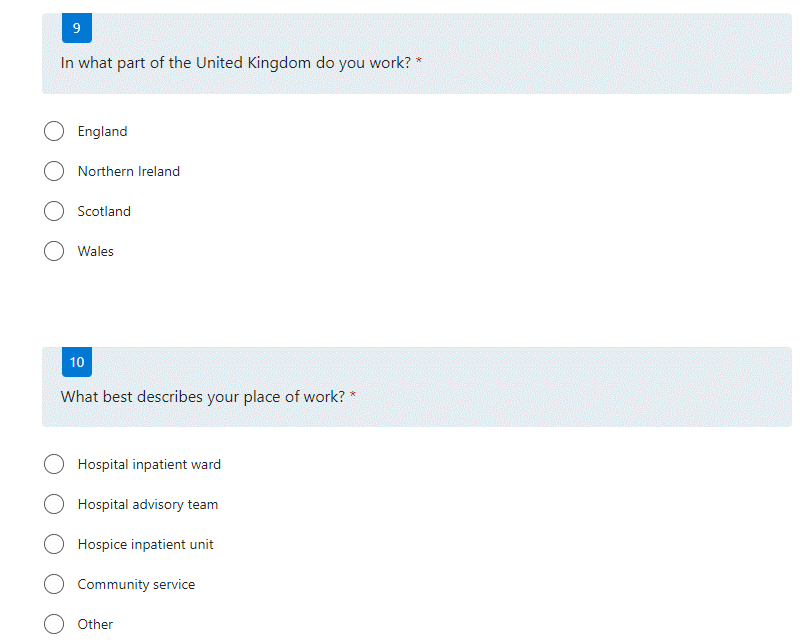


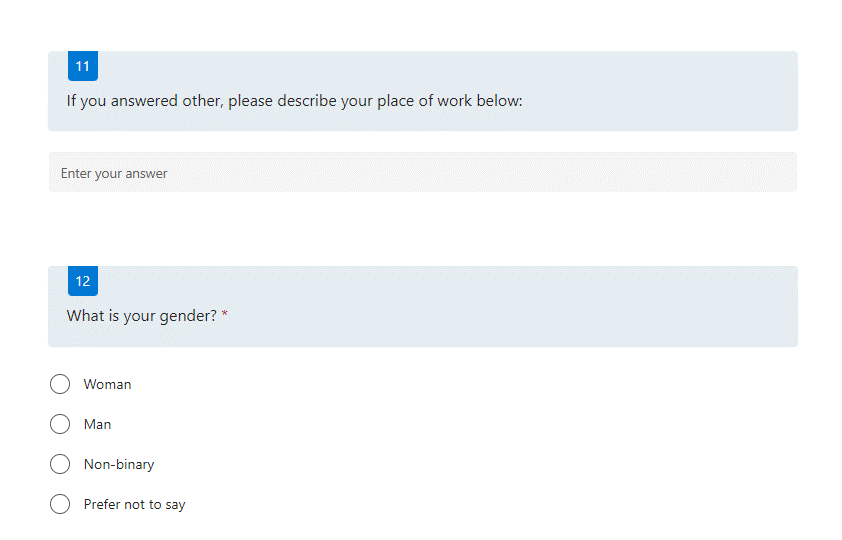


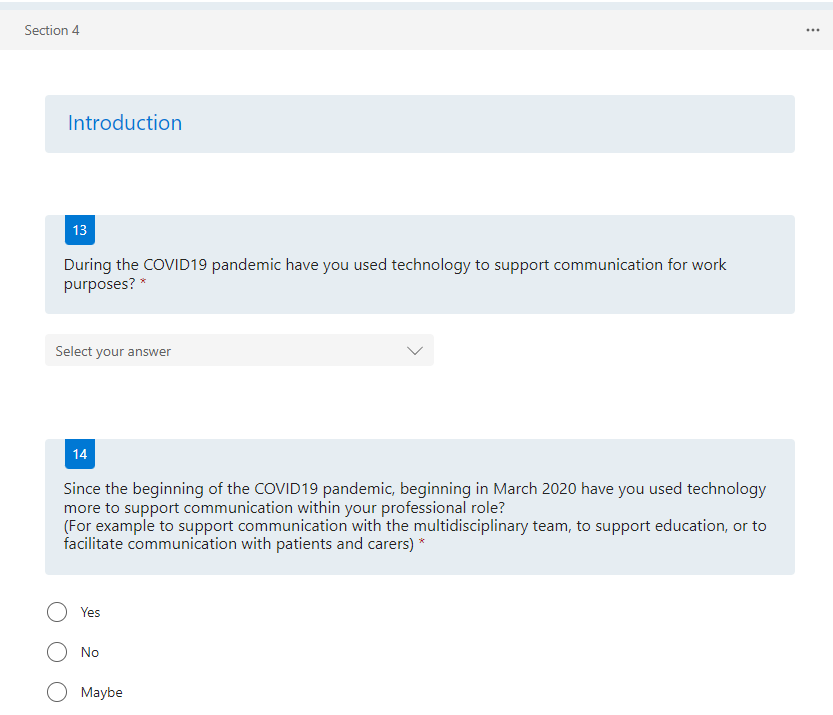


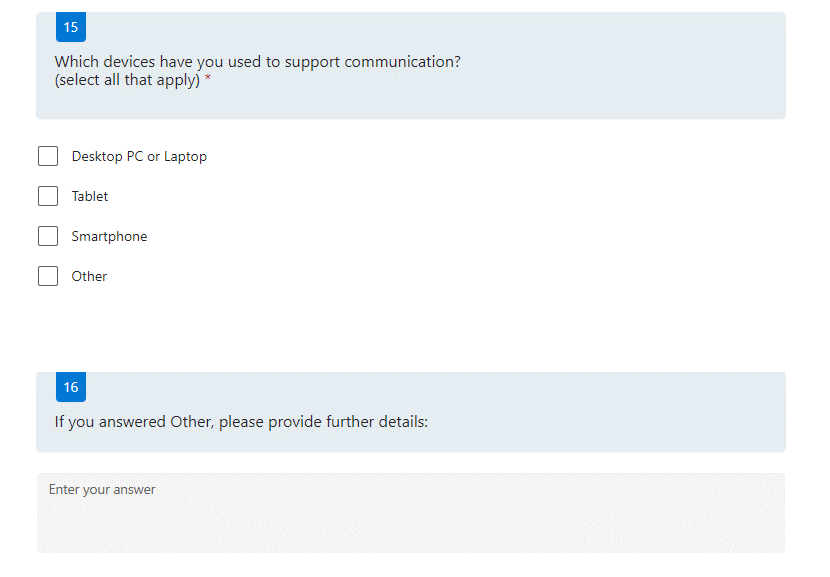


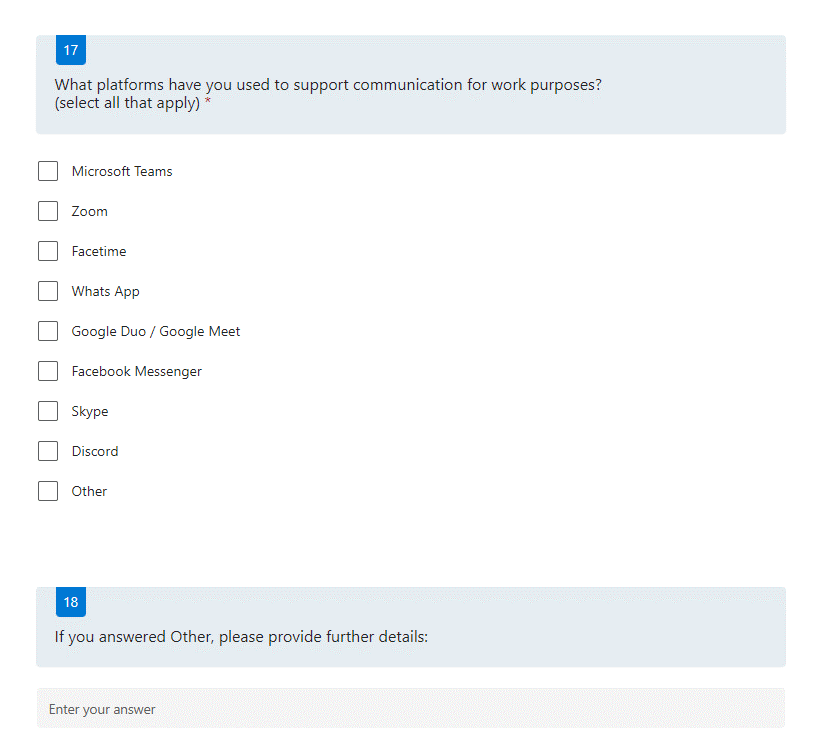


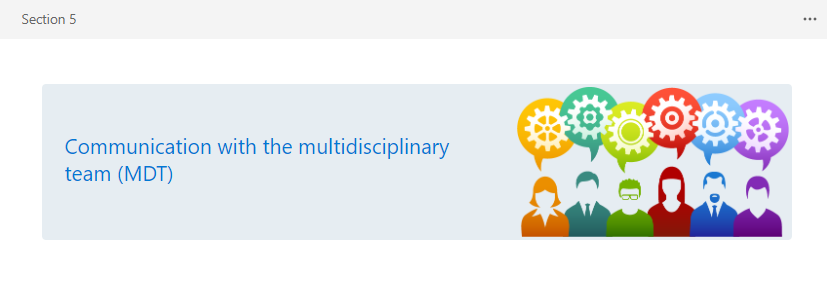


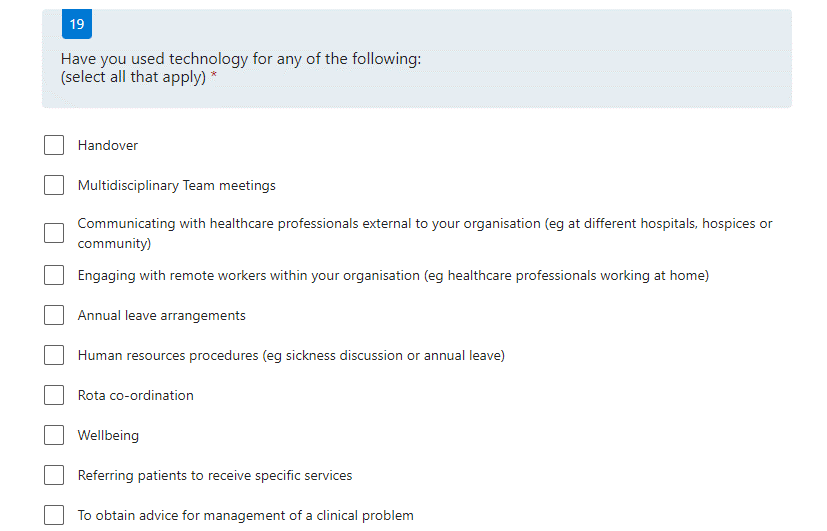


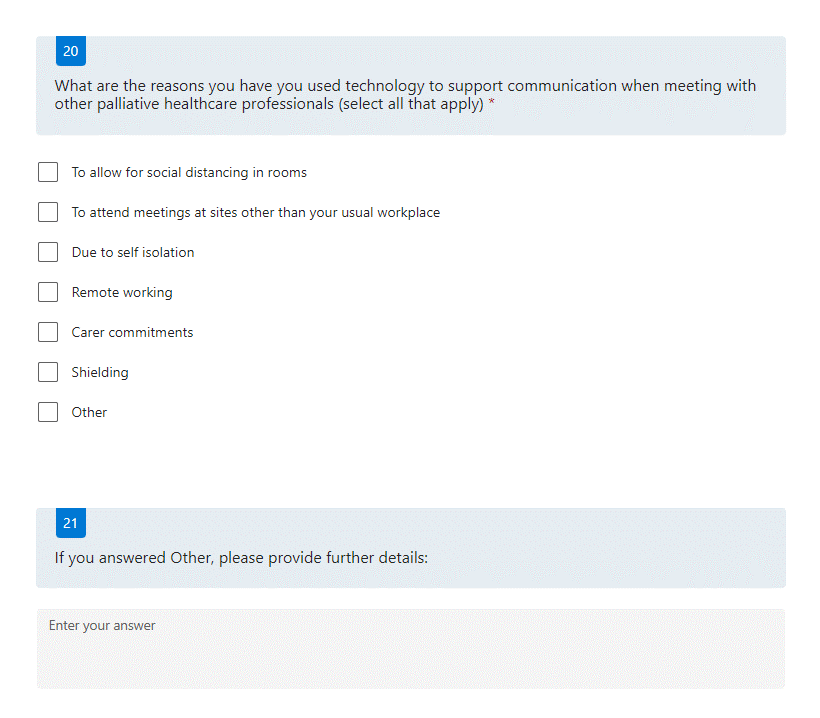


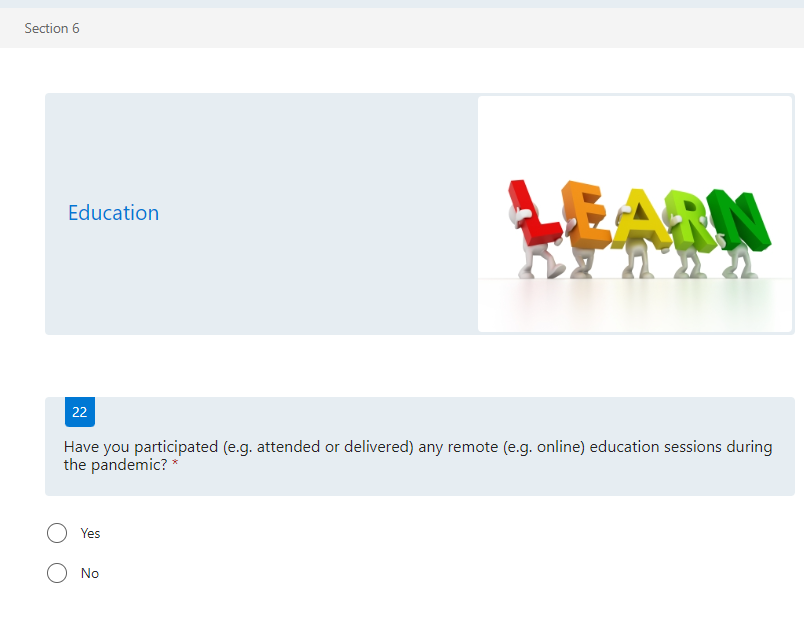


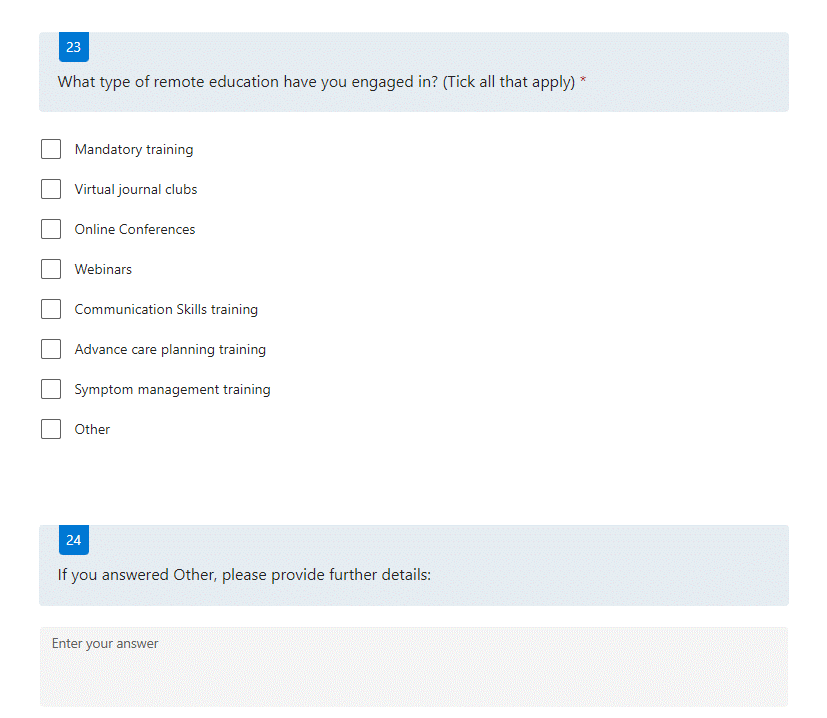


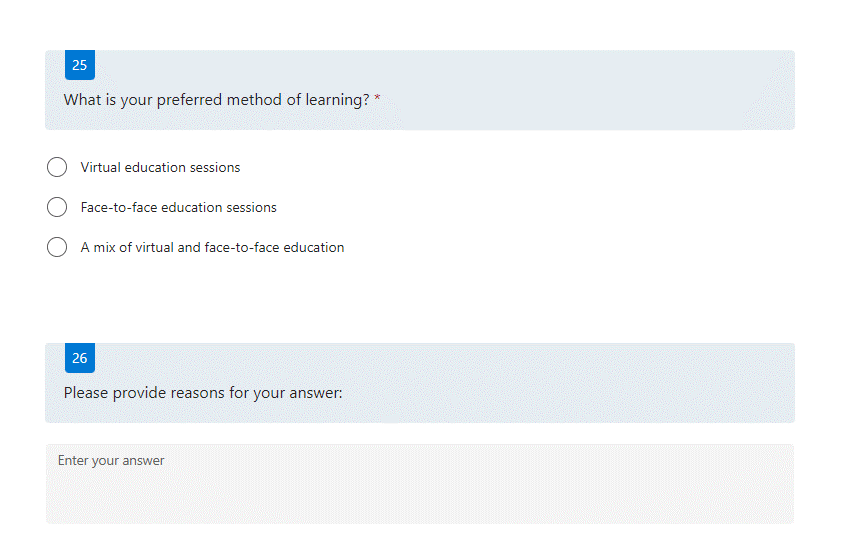


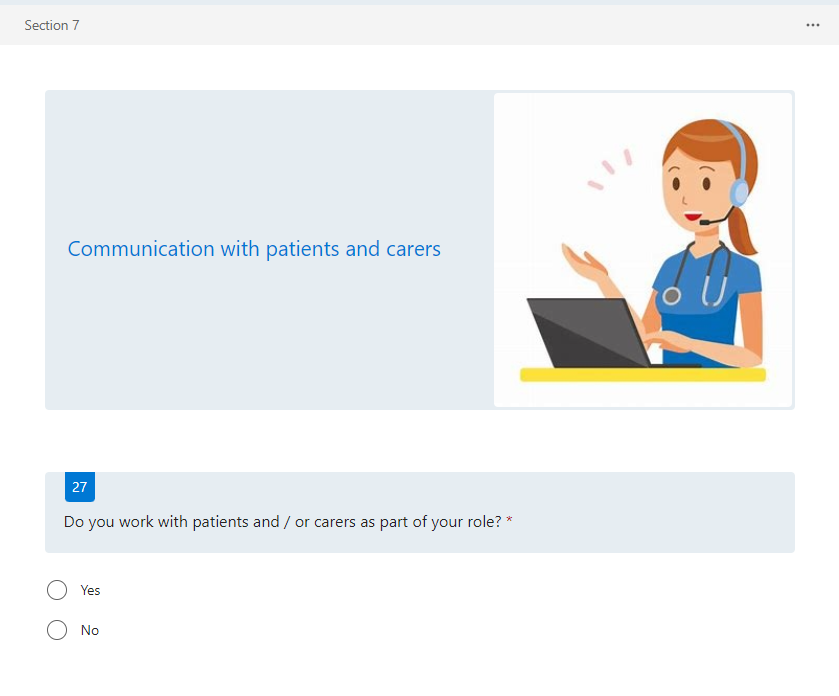


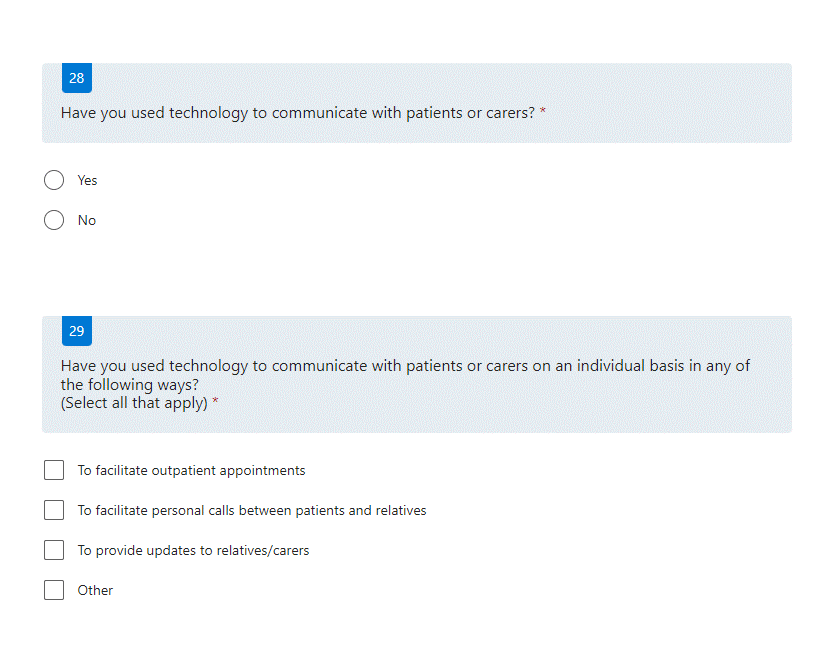


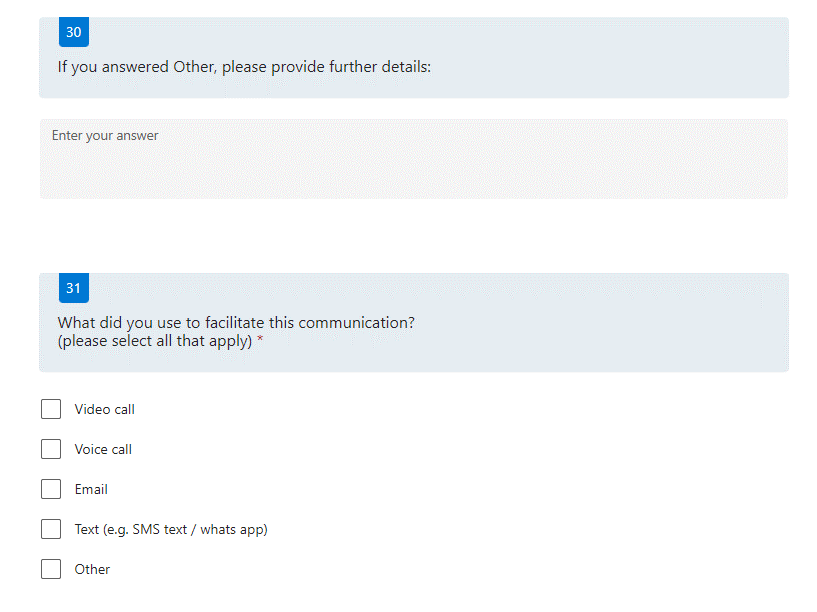


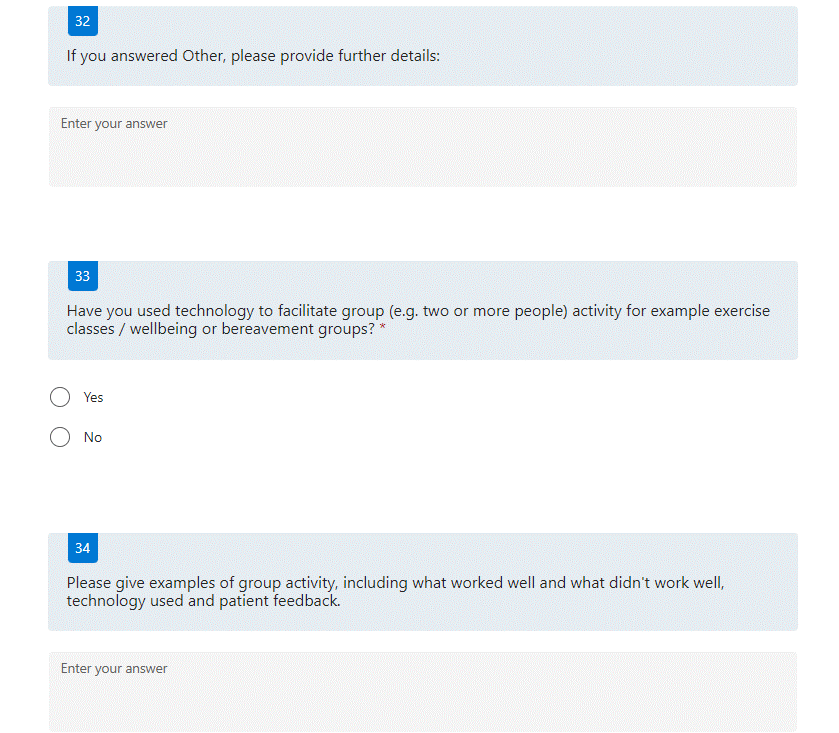


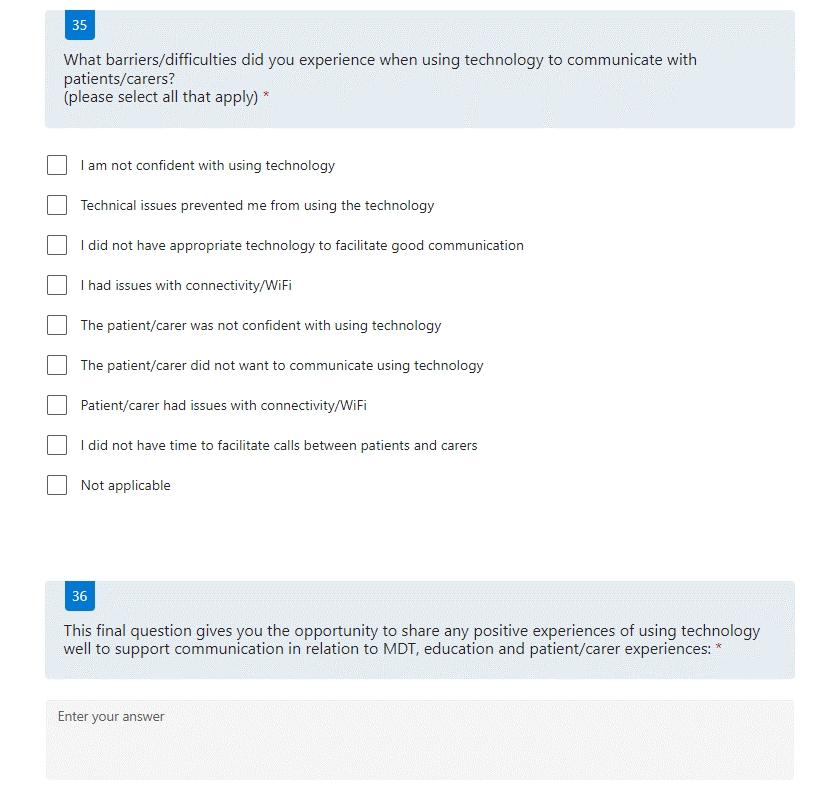


**Appendix 3. Timeline of survey dissemination**

05/05/21 – survey launched via social media accounts of SS and AN, emailed internally to Marie Curie Hospice Liverpool (MCHL) and study adverts placed around MCHL

05/05/21 - email to all research leads and hospice managers at other Marie Curie hospices asking to distribute survey

05/05/21 - Tweeted on Marie Curie EOLC twitter account

05/05/21 - Tweeted on Palliative Care Research Society twitter account

07/05/21 - Survey sent out to relevant Association of Palliative Medicine members

10/05/21 - emailed to CHAIN (contact, help, advice and information network)

10/05/21 - emailed to Marie Curie Nursing Service North West

14/05/21 - sent out to Marie Curie Belfast, Edinburgh, Glasgow, West Midlands hospices via research leads and nurses

21/05/21 - Email sent out on CHAIN network

26/05/21 – Tweeted via SS twitter account

05/06/21 - Survey sent out to relevant Association of Palliative Medicine members

07/06/21 - Tweeted via SS twitter account

17/06/21 - Tweeted via SS twitter account

30th June – Survey closed

**Appendix 4. Participant information sheet**

**How can technology be used to support communication in palliative care beyond the COVID-19 pandemic?**

**PARTICIPANT INFORMATION SHEET**

**Introduction**

We have provided you with this information, as we would like to invite you to consider taking part in a research study that we are currently undertaking. In order for you to decide whether you wish to take part, this information sheet has been created to explain why the research is being done and what it would involve for you, if you decide to take part. If after reading this information sheet, you have any questions about the study and wish to contact the research team, please feel free to do so using the contact details at the bottom of this information sheet.

**What is the purpose of the study?**

The COVID19 pandemic has presented our society with a lot of challenges. We have seen many changes to our healthcare systems, with one of the most notable being around how healthcare professionals are able to communicate with patients, carers and other professionals. The pandemic has highlighted how healthcare professionals can use technology to support patients and their families. An example of palliative care technology is video-calling technologies.

There is great potential to better use technology in palliative care. In order for us to do this we need to understand how technology has been used well, enabling development in the use of technology to improve care.

The aim of this research is to conduct a nationwide survey of healthcare professionals to identify how technology has been used to support communication in palliative care during the COVID19 pandemic. The survey will focus on the following themes and areas:

- Use of technology in handovers
- Use of technology in education
- Use of technology to support patients and carers

This project looks to identify learning beyond the pandemic, identifying knowledge and skills required to support healthcare professionals to adopt technology to support communication.

**Why have I been approached?**

You have been approached as you have been identified as a Palliative Healthcare professional working in hospital or community based healthcare during the COVID-19 pandemic beginning in March 2020.

**Do I have to take part?**

No. It is up to you to decide whether or not you wish to take part. We would like to reassure you also, that even if you have signed the consent form, you can withdraw from the study at any point, without giving a reason or an explanation and without repercussion.

**What will happen if I take part?**

We will invite you to take part in a questionnaire. The questionnaire will come as a ‘Microsoft Form’ which you will be able to complete and submit online. Your answers will be treated confidentially.

*Consent to Participate:*

Before deciding if you wish to take part in this questionnaire, please make sure you have read and are happy with all the information in this leaflet.

*Consent Form*:

A consent form will appear on the first page of the questionnaire, prior to completion. Your email address will be required in order to complete the consent form and begin the questionnaire. Your information will be kept strictly confidential and will be used for the purposes of this project only.

Please find the Microsoft forms link below: <https://forms.office.com/r/0c49i9gFS0>

**What will happen if I don’t want the study to carry on?**

If you change your mind about taking part, even after you have signed the consent form, you are free to withdraw from the study at any time without giving a reason or explanation. Any information we have collected from you prior to withdrawal will still be included in the study.

**Expenses and Payments**

You will not receive expenses for participation in this study.

**What are the possible disadvantages and risks of taking part?**

We do not think that there are any major risks involved in being part of this research. However, if at any point you have any concerns about any stage of the research study, or you experience any distress or upset as a result, you can contact the Principle Investigator (PI) for this study (contact details below), who will discuss this with you further.

**What are the possible benefits of taking part?**

There will likely be no direct benefit to you as a participant in this study. Your participation this study will however inform future research and policy for the use of technology to improve communication in palliative care, which will potentially lead to benefits for many people in the world living with serious illness.

**Will my taking part in the research be kept confidential?**

Yes. We will follow current ethical and legal practice and all information collected will be handled in confidence. With your permission, we would like to collect and store the information you provide as part of the study, for analysis. Data from completed questionnaires will be stored in a password protected secure electronic database to aid analysis. You will not be identified by name at any stage – only a number will be used, and only members of the research team will have access to the information you provide. The questionnaire data will be stored on a secure network drive for a period of 10 years after which it will be securely destroyed. You will not be named in any reports or published articles. Any paper questionnaires or analysed materials will be kept in a locked filing cabinet in the Marie Curie Hospice Liverpool research office, for 10 years after which they will be securely destroyed.

**What will happen to the results of this study?**

The outcomes of this study will look to develop recommendations for organisations of how technology can be used to improve communication in palliative care. These recommendations will provide guidance on how to support the adoption of technology, identify training needs, identify future uses for technology in supporting communication and provide wider learning for the future.

We hope that these recommendations will be used by organisations to make changes in digital communications in these three themed areas:

- Handover and MDT
- Education
- Patient/Carer interactions

We intend to publish the findings in professional journals and make presentations to national and international research conferences to make sure that the messages from this study are shared widely and appropriately.

**Who has reviewed the study?**

This proposal has been reviewed and approved by the University of Lancaster Research Ethics Committee,whose task it is to make sure that research participants are protected from harm. You can find out more about the work of Research Ethics Committees by visiting the National Research Ethics Service website at http://www.nres.npsa.nhs.uk.

**Further Information**

If you would like any further information about this study please contact:

**Principle Investigator**: Sarah Stanley

**Telephone**: 0151 801 1490

**Email**: sarah.stanley@mariecurie.org.uk

**Research Lead/Supervisor**: Dr Amara Nwosu

**Telephone**: 0151 801 1490

**Email**: a.nwosu@lancaster.ac.uk
